# Supplementary material for: Patterns of Intron Gain and Loss in Fungi
Source: PLoS Biol. 2004 Nov 30;2(12):e422. doi: 10.1371/journal.pbio.0020422 (PMC532390; doi:10.1371/journal.pbio.0020422)
Supplement: Table S1 — Also available at http://genes.mit.edu/NielsenEtAl/. (4.3 MB ZIP). [file pbio.0020422.st001.zip › NielsenEtAl/html/178.html]

AN7465.1.NCU03291.1.MG09891.1.FG00509.1


```
 CLUSTAL W (1.82) Multiple Sequence Alignments - Introns Inserted


Sequence 1: NCU03291.1	658 aa
Sequence 2: MG09891.1	625 aa
Sequence 3: FG00509.1	623 aa
Sequence 4: AN7465.1	584 aa
Alignment Length: 669 aa
Number Identitical Residues: 169 aa
Alignment Score (without introns) 9432


MG09891.1 	MFYSDTLLRTTGPLSRVWLAANMERKLSKAHILQSNLRHSVEQIIQPSEEAPLALRLSGQ
NCU03291.1	MFWSGTLLSATGPLAKAWLSANQERKVSKVQILQHNLQDSVDAIIAPN-DAPLALRLSGQ
FG00509.1 	MFYSEALLQKSGPLARVWLSANLERKLSKTHILQSNVADSVEAIIMPN-QAPMALRLSSQ
AN7465.1  	MFYSETLLSKTGPLARVWLSANLERKLSKSHILQSDIESSVSAIVDQG-QAPMALRLSGQ
          	**:* :**  :***::.**:** ***:** :*** ::  **. *:  . :**:*****.*

MG09891.1 	LLLGVVRIYSRKARYLLEDCNEALMKIKM0AFRSSGNNDLPTNLHLPNRESLMLQDRITP
NCU03291.1	LLLGVVRIYSRKARYLLDDCNEALMKIKM0AFRSTGNHDIPTNLHVQNSESLMLPDQITP
FG00509.1 	LLLGVVRIYQRKTRYLLDDCNEAMMKIKM0AFRSSGNNDMAVNLQIPNREALTLPDKITP
AN7465.1  	LLLGVVRIYSRKARYLLDDCNEALMKIKM0AFRLTNNNDLTTSAVVAPG-GITLPDVLTE
          	*********.**:****:*****:***** *** :.*:*:...  :    .: * * :* 

MG09891.1 	HDNLDDLPPE----PTLNFDDLTTSQQLGRKG--RVSNREINLQEDFNNSQFLQDESRNE
NCU03291.1	YDNLDLLPPPSADYLASQIEEVTGAPIMARKVQVRASQRDINLQEDFNNSQFLNNTMIDE
FG00509.1 	YDNFELPPPPDPNWLLSQVEDVTTAP-IGRKS--RVSQRDINLQEDYDNSQFLNDGMAMD
AN7465.1  	ADLFMNLDSS-----LLIPQPLSLEPEGKRPG----------PSMDFGSQLFPDTGLRRS
          	 * :    .          : ::      *             . *:... * :     .

MG09891.1 	LNLKVADMDED--LELDFGLDLD-----MDETVVETGRDAPAARDVEDDMISELVP--SK
NCU03291.1	EELALANNDDLG-LELDFGMDIDERPSKFMDTTIEMGRDAPNARAVEDDIFSELSAGPGK
FG00509.1 	DDMMAP-LGNID-LELDFGLDDLG----LEETGLEPEGGRREDRTAAGPDINDSELDFPQ
AN7465.1  	ASQEPALLEDPGDLQLNLGLDDETNLSFSHDFSMEVGRDAPAPRPMEEDNFSDAGKVIDV
          	 .   .   : ..*:*::*:*     :   :  :*   .    *      :.:       

MG09891.1 	ERDT---SLGLDFGDDEQVRIPDVEGDIPMFDDDMDFNIGDASAMPQALMEVAP---ERA
NCU03291.1	QRGTREPSLGLDLDYGDGVRIADDEGDIQMGDDDLQFNVADHSEIPEGLSTPGAPDMART
FG00509.1 	KDDQ-DRAMTLDLPRDD-VIIHDGGEDVVMEGMELDFNPDDVSQMPGIAGPEYP----PP
AN7465.1  	GDLG--LNLGEDDTPLDAVNFDANEDNFLPLDEPMDLG--DDTVVADGNDERFE------
          	        :  *    : * :     :.   .  :::.  * : :.              

MG09891.1 	RISESPLSDIDPAFANEMEMEFSRTMNGDMYEPGDDTERTITMKAPQRAKKRR---AIVP
NCU03291.1	RISESPLSDIDDEFAKEVEMEYSRHNNSDMYEPGEDPT-ASTIIAPQRSKKRK---LLQP
FG00509.1 	RISESPLSDIDEDRAAQLEEDHSRYQMADLYEPENEDD-QIVRRPAQRAKKQK---ILTP
AN7465.1  	--RESTLTEVSEDMIERLNTEHEG---DYMHDEEQDDE---TIQHAQRAKRRKQLPTIEL
          	   **.*:::.     .:: :..      :::  ::     .   .**:*:::. . :  

MG09891.1 	DTDTMLT-SNHIRDQQAHHDNILKPQTFVPNEPYLLTLLDMQKSGGFVSNVILESRSEEW
NCU03291.1	DEQTMLS-NAQIKEQQSKRDNILKPQSFGTQDPYLMALLDLQKNGAFVSSVLLENRSDSW
FG00509.1 	DEEIALS-SNHIKQQQANRDNIIKEAGFLPRDPFLLALMDMQKSGGFVSSIMGDKRSTNW
AN7465.1  	DEAVEFKGNSYFRIQQEQLSETLKPASFLPRDPVLLTLMNMQKNGDFVSNVMGGGRGRGW
          	*    :...  :: ** : .: :*   * ..:* *::*:::**.* ***.::   *.  *

MG09891.1 	APELKGLLSLESIRPINDLKRKRDSGIADMESGDDQGQSKSPRLELGEDDDQLN-LGGDD
NCU03291.1	ADELSDMLSLSTFRPF-DLKRKRDSAIADMEVDDEDGTRKSPRLELPDDESTVVGRAESI
FG00509.1 	APELRGILSLDTARGMNDLKRKRDSGIADVES--DHGANKSPRMLDIGSDNGLG--FEGG
AN7465.1  	APEIRDLLSFDTVRKAGELKRKRDSGISDMDVD----AAAAPALEIEEEAIVPV---DEG
          	* *: .:**:.: *  .:*******.*:*:: .       :* :    .           

MG09891.1 	SADQTVRPEGTVLEIPADEG-----YEQHDDTGYENVT-AMPAFDDTVAPQTLPADSGPI
NCU03291.1	NGNQSETAEGTEIELAANDDGAVFHDEDQERPGSRNANSPVPNFDETIAPIVHPADSGPV
FG00509.1 	FGDMSLPPDESRHEIPAEDE-------MDDDEG----ATMMPGFEDTTIALVHPAESGPV
AN7465.1  	VGMESTLHQRSEIDFPGDEED---HLRLSDDEG---AQQPLEDFDDTIT----PVDSALV
          	 .  :   : :  ::..:: .        :  *       :  *::*      *.:*. :

MG09891.1 	SIGTKHAVHILRDLFGPDAATDAEKRKNTSVVFQELLPETRTTKADATKMFFECLVLATK
NCU03291.1	SIGTKHAVHILRDLFGAEAANNADKRKKTAVVFQDLLPEGRTTKADATKMFFECLVLATK
FG00509.1 	SLGTKHVVHQLRDRFGDDAADSPGKRNERAVLFQNLCPRQEATKADATKMFFECLVLATK
AN7465.1  	SVGTKRAVHVLRDCLG-----NAEQKK--AVKFQDLLPEKKATRADATKMFFEVLVLATK
          	*:***:.** *** :*     .. :::  :* **:* *. .:*:********* ******

MG09891.1 	DAIKVEQPEGVLGGPIRIRGKRGLWGAWAEREAGGEMAAE---VDEPEPAVTSAPVVSVE
NCU03291.1	DAIKVEQKEGALGGHIRIRGKRGLWGDWAEREAGGEMAQQ---SQQQQPINPDEPQPSIE
FG00509.1 	DAIKVEQSSG-LGDDIRIRAKRGLWGAWAEREAGGEMSQDNDVSDEPEHAAAVTPAVAVS
AN7465.1  	DAVQVEQRSNTVGGPIKISGKRSLWGQWAEEDATGEVSQA-----------------QVA
          	**::*** .. :*. *:* .**.*** ***.:* **::                    : 

MG09891.1 	A---------
NCU03291.1	TAAQPIEASA
FG00509.1 	A---------
AN7465.1  	A---------
          	:
```
